# Transcatheter Closure of a Patent Ductus Arteriosus Using a Piccolo Duct Occluder

**DOI:** 10.7759/cureus.28226

**Published:** 2022-08-21

**Authors:** Pranjit Deb, Anindya Benerjee, Tapas Som, Ramachandra Barik

**Affiliations:** 1 Cardiology, All India Institute of Medical Sciences, Bhubaneswar, Bhubaneswar, IND; 2 Neonatology, All India Institute of Medical Sciences, Bhubaneswar, Bhubaneswar, IND

**Keywords:** amplatzer piccolo occluder, transcatheter closure, congestive heart failure, patent ductus arteriosus, low birth weight, premature

## Abstract

Transcatheter closure of patent ductus arteriosus (PDA) is feasible in low-birth-weight infants. A female baby was born prematurely with a birth weight of 924 g. She had a PDA measuring 3.7 mm. She was dependent on positive pressure ventilation for congestive heart failure in addition to the heart failure medications. She could not be discharged from the hospital even after 79 days of birth, and even though her weight reached 1.9 kg in the neonatal intensive care unit. We attempted to plug the PDA using an Amplatzer Piccolo Occluder, but the device failed to anchor. Then, the PDA was plugged using a 4-6 Amplatzer Duct Occluder using a 6-Fr sheath which was challenging.

## Introduction

Although surgical ligation of the patent ductus arteriosus (PDA) via a left thoracotomy has a remarkably high success rate, it is associated with the post-ligation syndrome, which includes pneumothorax, bleeding, accidental ligation of the left pulmonary artery or aorta, vocal cord palsy, and chylothorax. The reported delayed complications associated with surgical ligation of PDA are scoliosis and restrictive lung disease. Transcatheter closure (TCC) of PDA results in rapid improvement and less often is associated with post-ligation syndrome [1]. TCC of PDA in extremely-low-birth-weight (ELBW) infants is a feasible alternative to surgical ligation when the patient has persistent heart failure despite adequate medical treatment and positive pressure ventilation [2-5]. Complications are observed in less than three out of eleven cases during TCC of PDA using Amplatzer Piccolo Occluder (APO). The incidence of morbidity and mortality associated with these complications is extremely high because of frailty. Left pulmonary artery stenosis and device deformation have been reported after TCC of PDA using APO because of the relatively large-sized device [4]. Iatrogenic coarctation has been also reported in some cases which either resolves spontaneously with time or needs surgery after deployment of APO [6-8]. Sometimes, there may be embolization of APO into the pulmonary artery or aorta which needs surgical intervention [9,10]. Procedural complications can be minimized by adequate training and using a proper device [5,11-13].

## Case presentation

**Figure 1:**
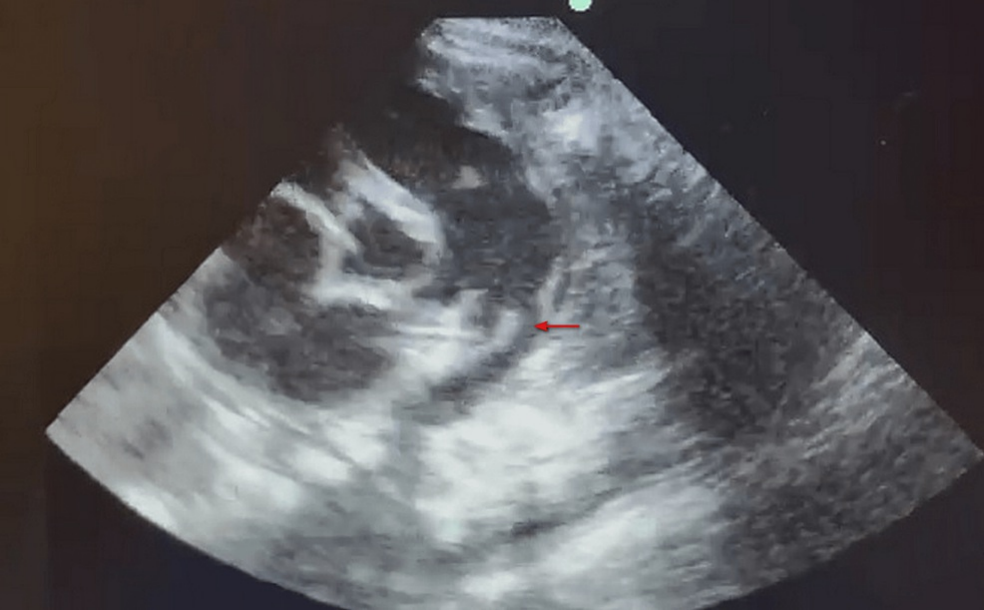
Two-dimensional transthoracic echocardiogram showing the patent ductus arteriosus. The echocardiogram showing the patent ductus arteriosus measuring 3.7 mm, as seen in the parasternal short axis view marked by a red arrow.

**Figure 2:**
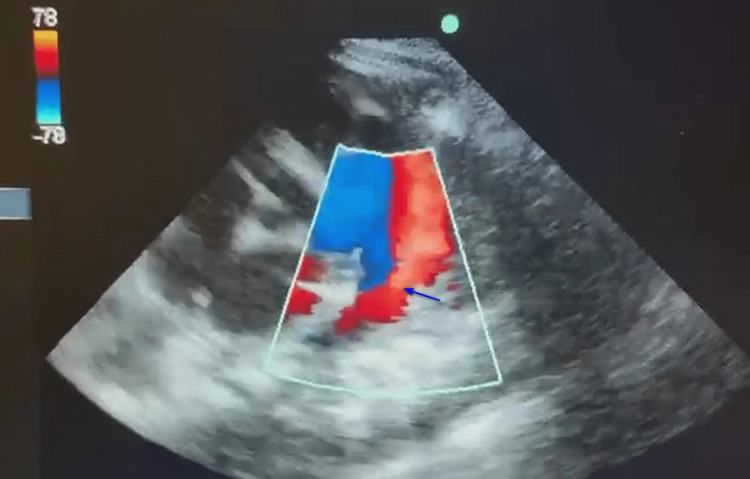
Color Doppler of the patent ductus arteriosus. Color Doppler of the patent ductus arteriosus showing predominantly flowing left to right shunt (only red flow), as seen in the parasternal short axis view marked by a deep blue arrow.

A premature female baby was born at 29 weeks with a birth weight of 924 g in an outside hospital. She was referred to us for congestive heart failure (CHF). She had significant left to right shunt due to a 3.7 mm PDA evaluated by transthoracic echocardiography (Figures 1, 2).

The neonate was admitted to the neonatal intensive care unit (NICU) for medical stabilization using continuous positive airway pressure (CPAP), repeated doses of ibuprofen, and loop diuretic. She weighed 1.9 kg when she was 79 days old during her NICU stay. TCC of PDA was considered as the infant could not be discharged because of CHF caused by a significant left to right shunt through the PDA. The management of the case was discussed with the faculty from cardiovascular surgery, cardiology, and cardiac anesthesia for surgical ligation versus TCC. The parents of the infant were counseled. The parents gave consent for TCC of PDA. The size of the femoral vein and the femoral artery of the baby was 3.6 mm and 2.1 mm, respectively. The planned TCC of PDA using the APO (Abbott Structural Heart, Plymouth, MN, USA) from the pulmonary arterial end was decided under general anesthesia and mechanical ventilation support [14].

**Figure 3:**
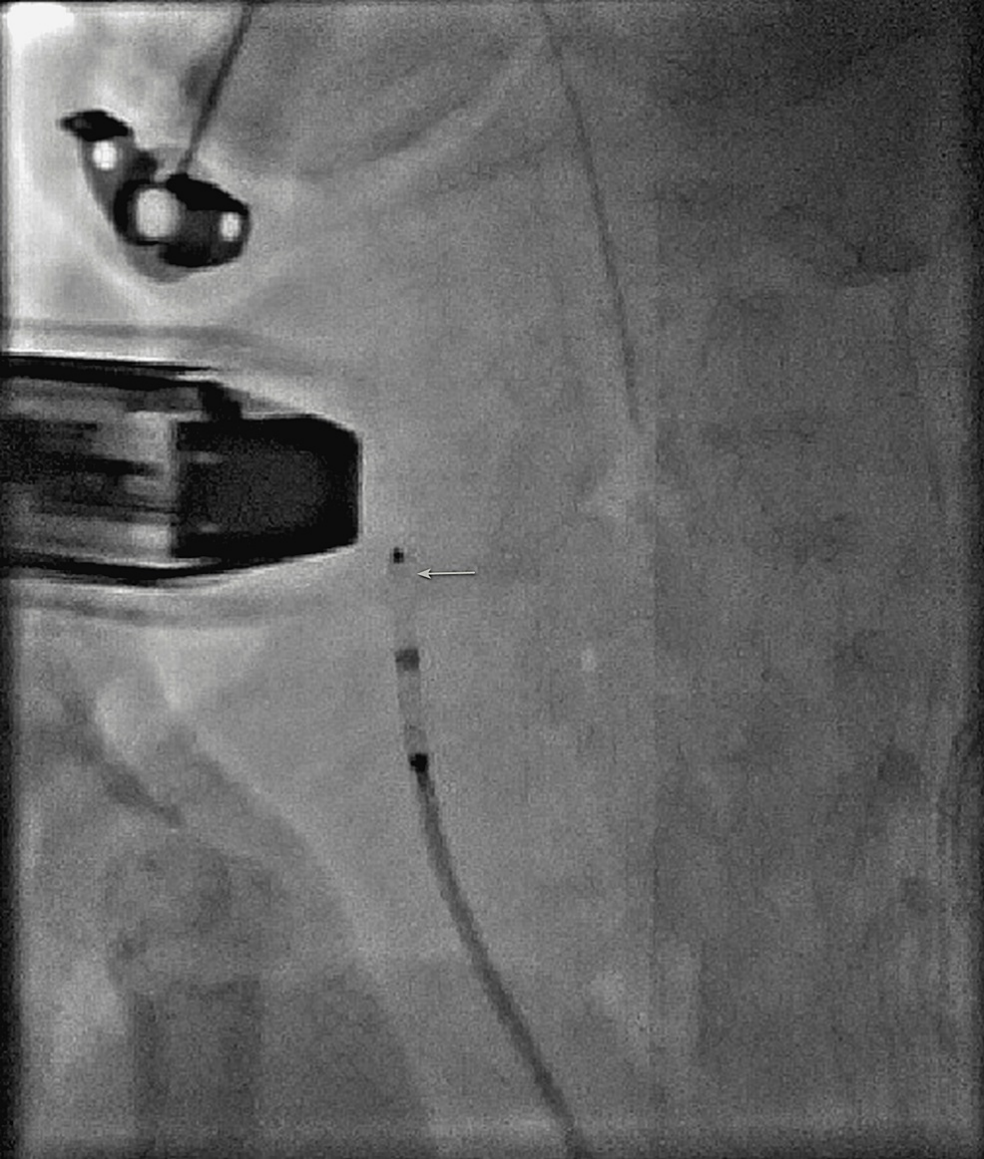
Amplatzer Piccolo Occluder implantation. Fluoroscopy in the 90-degree lateral projection showing that the Amplatzer Piccolo Occluder implantation failed to anchor the duct, as marked by the yellow arrow.

The PDA was crossed from its pulmonary arterial end under the guidance of fluoroscopy and echocardiography. Based on the echocardiographic assessment, the PDA measured 3.7 mm at its narrowest diameter (Figure 1). A 05-04 APO was chosen through a 4-Fr delivery sheath. However, the device failed to anchor at the defect (Figure 3).

**Figure 4:**
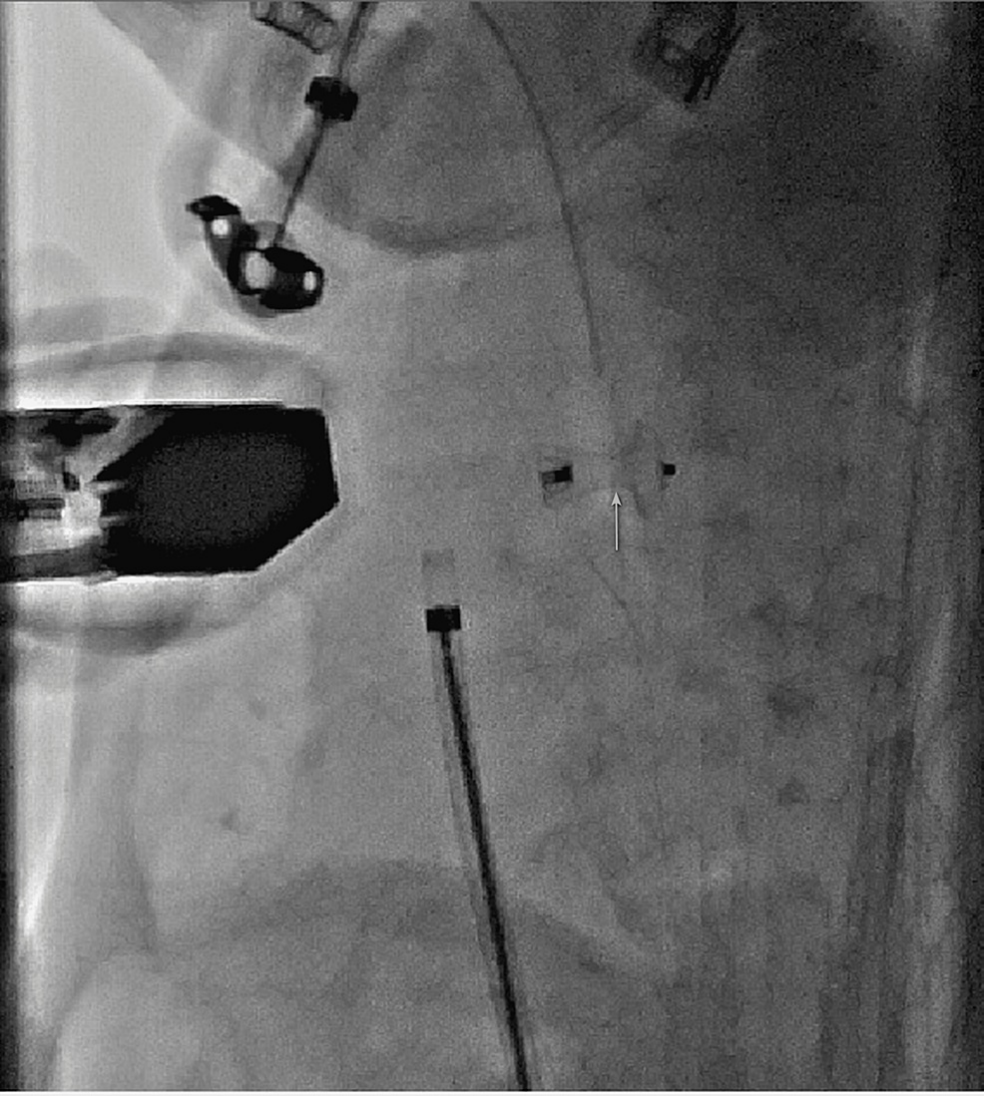
Amplatzer Duct Occluder implantation. Fluoroscopy in the 90-degree lateral view showing successful deployment of a 04-06 Amplatzer Duct Occluder with no residual shunt, as marked by a yellow arrow.

Next, a needed higher size APO (05-06) was not available at this time. We eventually deployed a 04-06 size Amplatzer Duct Occluder through a 6-Fr delivery sheath under the guidance of fluoroscopy and 40 echocardiography (Figure 4).

Post-deployment angiography in the lateral view showed that the PDA device was snuggly fitting the defect without any residual shunt. There was no obstruction to the pulmonary artery or the descending thoracic aorta. The negotiation of the 6-Fr delivery sheath was difficult at the groin and across the PDA. Mild pericardial effusion was noticed immediately after the deployment of the PDA device. The pericardial effusion did not progress on subsequent evaluation and follow-up. The femoral vein sheath contrast injection showed some haziness around the iliac vein which also did not progress on subsequent follow-up. At 15 hours post-procedure, the infant suddenly started desaturating and sustained bradycardia. Echocardiography showed the duct occluder in place and there was a thin rim of pericardial effusion at this time. Resuscitation was tried but the infant could not be revived. Despite the use of reasonable procedural time, technique, less contrast, and low blood loss, we do not know the exact cause of death in this infant. A post-mortem could have helped us to diagnose the exact cause, but the parents did not agree to a pathological autopsy.

## Discussion

The APO device was approved by the Food and Drug Administration (FDA) in 2019 for usage in premature infants weighing >700 g and aged more than three days. A prospective multicenter study of 200 patients, which included 100 patients weighing less than 2 kg showed that the Piccolo device could be used safely in infants weighing more than 700 g [4]. An APO is a preferred choice for the TCC of a PDA in neonates owing to its smaller device profile when compared to an ADO, which allows it to be delivered through a 4-Fr delivery system. An ADO requires a minimum of 6-Fr delivery system which is relatively larger for neonates of any size. A 6-Fr delivery system required for an ADO can injure the femoral vein, iliac vein, and the junction of the inferior vena cava and right atrium and PDA. The retrieval of ADO is more challenging when embolized because of its larger size. Although there is no head-to-head comparison between the ADO and APO, there are many technical advantages of the latter device, which makes it the preferred choice of device for TCC in neonates whereas the ADO device is recommended by the manufacturer to be used in infants above 5 kg body weight [15]. Our patient was considered for device closure on day 79 of life and weighing 1.9 kg at the time. The initial attempt with 05-04 APO failed as the device failed to anchor to the PDA. Because a larger-sized APO 05-06 was not available at the time, an ADO device was tried. In an APO, both the aortic and pulmonary anchoring discs are of the same size unlike that of the ADO device where only the aortic end has a retention disk that measures larger than the pulmonary disc. Our patient had a large aortic ampulla which favored deployment of an ADO. The PDA was successfully plugged with a 04-06 ADO device. This is an unusual bail-out situation but not without certain drawbacks, such as minor femoral vein injury and mild pericardial effusion just after the procedure. This patient succumbed after 15 hours of the procedure.

## Conclusions

APO has revolutionized the TCC of a high-flow PDA in low-birth-weight infants because the device has a small profile and can be negotiated through a 4-Fr sheath which matches the diameter of the femoral vein in most neonates. The selection of an appropriately sized device, a proper technique, reducing procedural time, less contrast, low blood loss, and immediate attention to the possible complications are crucial for a successful outcome in low-birth-weight neonates. Our case depicts a special instance where a 04-06 ADO was used to plug a 3.7 mm PDA when a 04-05 APO failed to anchor the PDA in a low-birth-weight preterm baby but negotiation of the delivery sheath was a challenge.
